# Progress Toward Achieving National HIV/AIDS Strategy Goals for Quality of Life Among Persons Aged ≥50 Years with Diagnosed HIV — Medical Monitoring Project, United States, 2017–2023

**DOI:** 10.15585/mmwr.mm7336a1

**Published:** 2024-09-12

**Authors:** Linda Beer, Yunfeng Tie, Stacy M. Crim, John Weiser, Jennifer Taussig, Jason A. Craw, Kate A. Buchacz, Ashanté Dobbs, Charles B. Collins, Marie E. Johnston, Andrew De Los Reyes, Deborah Gelaude, Kamal Hughes, Rodel Desamu-Thorpe, Joseph Prejean

**Affiliations:** ^1^Division of HIV/AIDS Prevention, National Center for HIV/AIDS, Viral Hepatitis, STD, and TB Prevention, CDC; ^2^Oak Ridge Institute for Science and Education, Oak Ridge, Tennessee.

**Summary**

**What is already known about this topic?**

The U.S. National HIV/AIDS Strategy set 2025 goals for improving quality of life among persons with diagnosed HIV (PWH), monitored through five indicators: self-rated health, unmet needs for mental health services, unemployment, hunger or food insecurity, and unstable housing or homelessness. Among the growing population of PWH aged ≥50 years, progress toward these goals has not been assessed.

**What is added by this report?**

By 2022, no 2025 goal was met for PWH aged ≥50 years. If recent trends continue, goals are unlikely to be met. Although no goal was met for PWH aged ≥50 years overall, the goal for reducing hunger or food insecurity was met for those aged ≥65 years.

**What are the implications for public health practice?**

Multisectoral strategies to improve access to housing, employment, food, and mental health could improve quality of life among PWH aged ≥50 years.

## Abstract

Ensuring good quality of life (QoL) among persons with diagnosed HIV (PWH) is a priority of the National HIV/AIDS Strategy (NHAS), which established 2025 goals for improving QoL. Goals are monitored through five indicators: self-rated health, unmet needs for mental health services, unemployment, hunger or food insecurity, and unstable housing or homelessness. Among the growing population of PWH aged ≥50 years, progress toward these goals has not been assessed. Data collected during the 2017–2022 cycles of the Medical Monitoring Project, an annual complex sample survey of U.S. adults with diagnosed HIV, assessed progress toward NHAS 2025 QoL goals among PWH aged ≥50 years, overall and by age group. The recent estimated annual percentage change from baseline (2017 or 2018) to 2022 was calculated for each indicator. Among PWH aged ≥50 years, the 2025 goal of 95% PWH with good or better self-rated health is 46.2% higher than the 2022 estimate. The 2025 goals of a 50% reduction in the other indicators range from 26.3% to 56.3% lower than the 2022 estimates. Decreasing hunger or food insecurity by 50% among PWH aged ≥65 was the only goal met by 2022. If recent trends continue, other NHAS QoL 2025 goals are unlikely to be met. Multisectoral strategies to improve access to housing, employment, food, and mental health will be needed to meet NHAS 2025 goals for QoL among older PWH.

## Introduction

**FIGURE 1:**
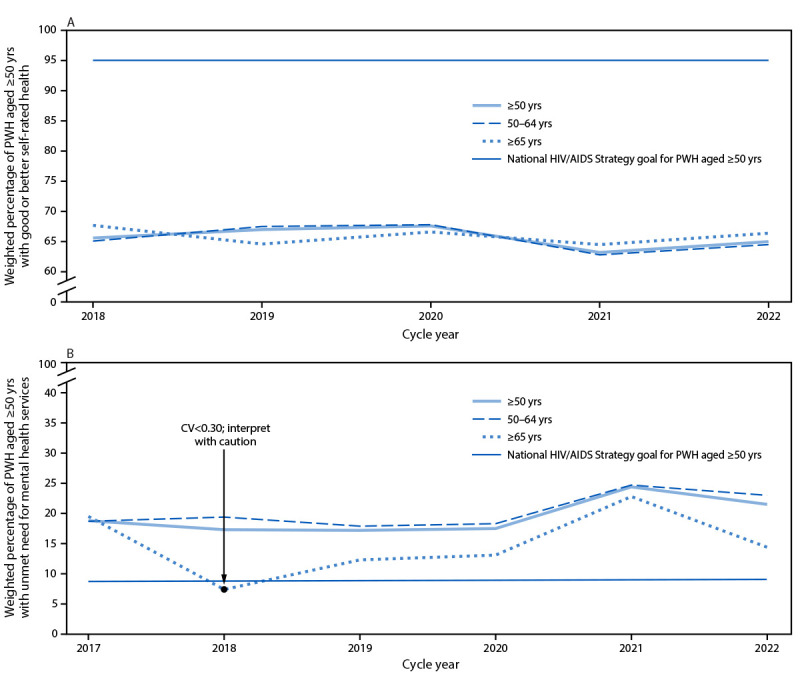
Trends in the weighted percentage of adults aged ≥50 years with diagnosed HIV with good or better self-rated health* (A) and unmet need for mental health services among those with any need for services^†^ (B), compared with National HIV/AIDS Strategy 2025 goals,^§^ overall and stratified by age group — Medical Monitoring Project, United States, 2017–2022^¶^ **Abbreviations:** CV = coefficient of variation; PWH = persons with diagnosed HIV. * PWH aged ≥50 years who reported their general health at the time of interview to be good, very good, or excellent as opposed to poor or fair. ^†^ PWH aged ≥50 years who reported needing but not receiving services from a mental health professional during the previous 12 months among all PWH aged ≥50 years receiving, or needing but not receiving, services from a mental health professional. ^§^ National HIV/AIDS Strategy 2025 goals for PWH aged ≥50 years are available online. https://files.hiv.gov/s3fs-public/2022-09/NHAS_Federal_Implementation_Plan.pdf ^¶^ Annual data collection cycles began June 1 of the cycle year and ran through May 30 of the following year. Collection of data on good or better self-rated health began in the 2018 cycle.

**FIGURE 2:**
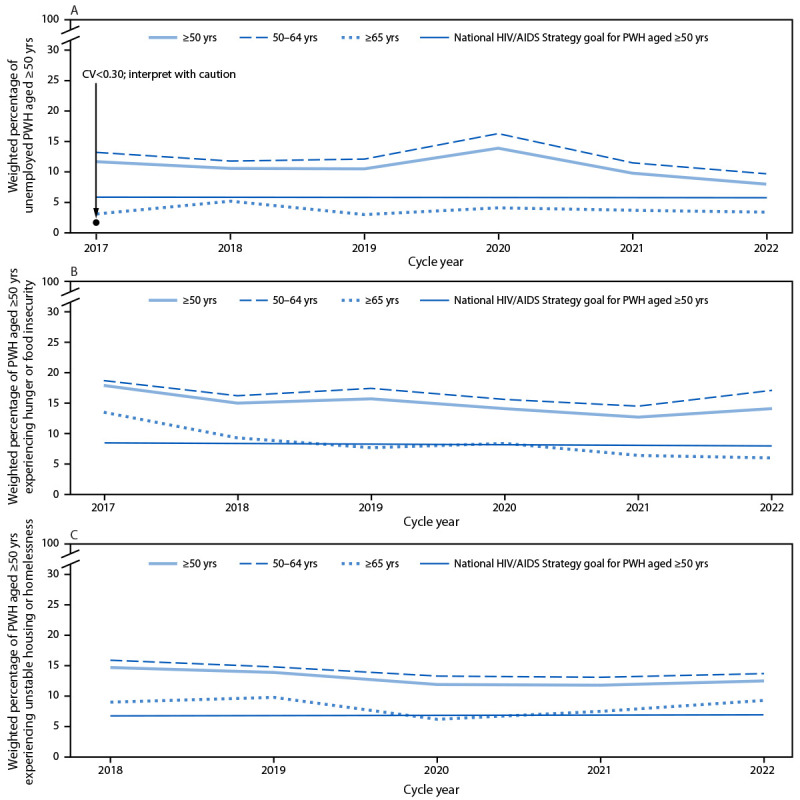
Trends in the weighted percentage of adults aged ≥50 years with diagnosed HIV who experienced unemployment* (A), hunger or food insecurity^†^ (B), and unstable housing or homelessness^§^ (C), compared with National HIV/AIDS Strategy 2025 goals,^¶^ overall and stratified by age group — Medical Monitoring Project, United States, 2017–2022** **Abbreviations:** CV = coefficient of variation; PWH = persons with diagnosed HIV. * PWH aged ≥50 years who reported being out of work at the time of interview as opposed to being employed for wages, a homemaker, a student, retired, or unable to work. ^†^ PWH aged ≥50 years who reported being hungry and not eating because of lack of money for food during the previous 12 months. ^§^ PWH aged ≥50 years who reported moving in with others because of financial issues, moving more than two times, being evicted, or living on the street, in a shelter, in a single-room–occupancy hotel, or in a car during the previous 12 months. ^¶^ National HIV/AIDS Strategy 2025 goals for PWH aged ≥50 years are available online. https://files.hiv.gov/s3fs-public/2022-09/NHAS_Federal_Implementation_Plan.pdf ** Annual data collection cycles began June 1 of the cycle year and ran through May 30 of the following year. Collection of data on unstable housing or homelessness began in the 2018 cycle.

As advances in HIV treatment have resulted in improved health and longevity (*1*), a large and growing proportion of U.S. persons with diagnosed HIV (PWH) are now aged ≥50 years (*2*). PWH are disproportionately affected by adverse social determinants of health, which affect their HIV-related health (*3*,*4*). To ensure good quality of life (QoL) among PWH, in 2022 the National HIV/AIDS Strategy (NHAS) set 2025 goals for improving five QoL indicators (*5*). These include 1) good or better self-rated health,* 2) unmet need for mental health services,^†^ 3) unemployment,^§^ 4) hunger or food insecurity,^¶^ and 5) unstable housing or homelessness.** Indicator goals are designed to increase good or better self-rated health to 95% and decrease all other indicators by 50% from their respective baselines by 2025. Baseline values and 2025 goals are presented in Figures 1 and 2 and in the Table. As persons age, their needs might change because of increasing age-related comorbidities and becoming eligible for Medicare. Thus, age-stratified estimates of QoL, and factors affecting QoL, among older age groups can help guide intervention strategies. QoL indicators are monitored using data from the Medical Monitoring Project (MMP) (*6*), a CDC-funded HIV surveillance system. This analysis examined recent trends in QoL indicators among PWH aged ≥50 years (overall and stratified by age 50–64 and ≥65 years), assessed whether recent trends are sufficient to meet NHAS 2025 QoL goals, and examined selected theoretically related factors potentially affecting the indicators (hereafter referred to as factors) to help guide intervention efforts to improve QoL among older PWH.

## Methods

### Data Collection

MMP uses a two-stage sample design: 1) 16 states and Puerto Rico were sampled from among all U.S. states, the District of Columbia, and Puerto Rico and 2) simple random samples of adult PWH were selected annually within participating jurisdictions from the National HIV Surveillance System (NHSS) (*6*). Interview and medical record abstraction data were collected in annual cycles during June 2017–May 2023. Annual response rates were 100% at the state and territory level and ranged from 40% to 46% at the PWH level. MMP was reviewed by CDC, deemed not research, and was conducted consistent with applicable federal law and CDC policy.^††^

### Statistical Methods

Data were weighted for unequal selection probabilities, adjusted for nonresponse, and poststratified to NHSS population totals. Among 13,475 PWH aged ≥50 years who participated in the 2017–2022 MMP cycles, weighted prevalence estimates and 95% CIs were calculated for each QoL indicator and theoretically related factors, overall and stratified by age (50–64 versus ≥65 years). For each indicator and theoretically related factor, Poisson regression models were used to calculate the recent estimated annual percentage change (EAPC) from baseline (2017 or 2018 cycle, depending on the indicator) to the 2022 cycle. EAPC measures the average percentage change per year over the period for which it is calculated. The percentage difference between the 2025 NHAS goal and the 2022 estimate, expressed as a percentage of the 2022 estimate, was also calculated (i.e., [2025 goal − 2022 estimate] / 2022 estimate).

## Results

### Good or Better Self-Rated Health

The 2025 NHAS goal for PWH aged ≥50 years to self-report good or better health is 95%. During 2018, 65.6% (95% CI = 63.5%–67.6%) of these adults reported good or better health and 65.0% (95% CI = 62.7%–67.2%) reported this in 2022. (Figure 1) (Supplementary Table 1, https://stacks.cdc.gov/view/cdc/160729). The 2025 goal is 46.2% higher than the 2022 estimate. Change in factors influencing self-rated health was minimal (Table) (Supplementary Table 2, https://stacks.cdc.gov/view/cdc/160728). Age-stratified trends were similar across the goal and factors that might influence it.

**TABLE:** Estimated annual percentage change in factors related to National HIV/AIDS Strategy quality of life indicators* among persons aged ≥50 years with diagnosed HIV, overall and stratified by age — Medical Monitoring Project, United States, 2017–2022^†^

Characteristic^§^	Age group, yrs	2017 cycle		2022 cycle		EAPC 2017 to 2022 cycles
		No.	Weighted % (95% CI)	No.	Weighted % (95% CI)	
**Factors related to good or better self-rated health** ^¶^						
Sustained viral suppression	≥50	1,593	68.9 (65.4 to 72.3)	1,614	67.3 (63.4 to 71.3)	−0.5 (−0.6 to −0.5)
	50–64	1,320	67.9 (64.5 to 71.3)	1,159	66.4 (62.0 to 70.9)	−0.8 (−0.9 to −0.7)
	≥65	273	74.2 (67.7 to 80.8)	455	69.8 (64.4 to 75.2)	−0.5 (−0.6 to −0.3)
Antiretroviral dose adherence, previous 30 days	≥50	1,396	66.5 (64.1 to 68.8)	1,515	71.8 (69.3 to 74.4)	1.9 (1.8 to 2.0)
	50–64	1,139	64.4 (61.7 to 67.2)	1,053	68.9 (66.0 to 71.7)	1.7 (1.6 to 1.8)
	≥65	257	77.4 (72.6 to 82.3)	462	79.7 (75.9 to 83.5)	1.2 (1.1 to 1.4)
Self-reported disability	≥50	1,155	52.8 (49.7 to 55.8)	1,037	48.0 (45.7 to 50.3)	−2.1 (−2.2 to −2.1)
	50–64	952	51.4 (48.3 to 54.4)	743	47.9 (45.3 to 50.4)	−1.9 (−2.0 to −1.8)
	≥65	203	60.4 (54.0 to 66.8)	294	48.4 (44.1 to 52.7)	−3.8 (−4.0 to −3.6)
Emergency department visit	≥50	849	38.7 (36.2 to 41.2)	802	36.5 (34.7 to 38.4)	−2.0 (−2.1 to −1.9)
	50–64	727	39.6 (36.9 to 42.2)	597	36.7 (34.5 to 38.9)	−2.2 (−2.4 to −2.1)
	≥65	122	33.8 (28.6 to 38.9)	205	36.0 (32.4 to 39.6)	−1.1 (−1.3 to −0.9)
Hospitalization	≥50	460	21.4 (19.5 to 23.2)	400	18.2 (16.3 to 20.1)	−3.7 (−3.9 to −3.6)
	50–64	378	20.8 (18.6 to 22.9)	284	16.8 (14.7 to 18.9)	−4.8 (−4.9 to −4.6)
	≥65	82	24.7 (19.8 to 29.5)	116	22.3 (17.5 to 27.1)	−1.9 (−2.2 to −1.6)
Factors related to unmet needs for mental health services**						
Symptoms of major or other depression among those with any mental health service need	≥50	263	33.9 (29.1 to 38.7)	211	27.0 (22.4 to 31.6)	−4.2 (−4.3 to −4.0)
	50–64	237	34.4 (29.3 to 39.5)	177	27.8 (23.1 to 32.4)	−4.0 (−4.3 to −3.8)
	≥65	26	29.9 (19.5 to 40.3)	34	23.3 (15.0 to 31.7)	−3.8 (−4.3 to −3.2)
Symptoms of generalized anxiety disorder among those with any mental health service need	≥50	218	27.0 (23.2 to 30.8)	200	25.3 (20.7 to 30.0)	−0.9 (−1.1 to −0.7)
	50–64	198	27.6 (23.7 to 31.5)	170	26.7 (22.0 to 31.4)	−0.2 (−0.5 to −0.0)
	≥65	20	22.3 (13.6 to 31.0)	30	19.1 (11.2 to 27.0)	−2.6 (−3.3 to −2.0)
**Factors related to unemployment** ^††^						
Some college education or higher educational attainment	≥50	1,230	55.6 (51.3 to 59.9)	1,300	60.3 (57.7 to 63.0)	2.0 (1.9 to 2.1)
	50–64	1,016	54.5 (50.5 to 58.4)	921	59.4 (56.7 to 62.1)	2.2 (2.1 to 2.3)
	≥65	214	62.1 (54.0 to 70.1)	379	63.0 (57.7 to 68.2)	0.2 (0.0 to 0.4)
Household income at or below poverty threshold	≥50	879	41.6 (36.3 to 46.8)	707	35.2 (31.0 to 39.4)	−3.8 (−3.9 to −3.7)
	50–64	758	42.4 (37.0 to 47.8)	538	36.8 (32.4 to 41.1)	−3.8 (−3.9 to −3.6)
	≥65	121	37.1 (30.3 to 43.9)	169	31.0 (25.5 to 36.5)	−2.4 (−2.6 to −2.1)
**Factors related to hunger or food insecurity** ^§§^						
Unmet need for food assistance or food stamps	≥50	243	11.8 (9.9 to 13.8)	211	10.4 (8.5 to 12.3)	−2.8 (−3.0 to −2.6)
	50–64	214	12.7 (10.7 to 14.8)	180	12.3 (10.0 to 14.7)	−1.1 (−1.3 to −0.9)
	≥65	29	6.9 (3.9 to 9.9)	31	5.0 (3.1 to 6.9)	−6.9 (−7.5 to −6.4)
Unmet need for food or meal delivery	≥50	161	7.6 (6.3 to 8.8)	160	8.3 (6.2 to 10.4)	−0.4 (−0.6 to −0.2)
	50–64	138	7.9 (6.5 to 9.3)	131	9.2 (6.7 to 11.7)	0.5 (0.2 to 0.8)
	≥65	23	5.7 (3.0 to 8.3)	29	5.9 (3.3 to 8.5)	−0.6 (−1.2 to 0.1)
**Factors related to housing instability or homelessness** ^¶¶^						
Unmet need for shelter or housing services	≥50	194	9.6 (8.0 to 11.2)	210	10.4 (8.4 to 12.3)	1.6 (1.4 to 1.8)
	50–64	178	10.3 (8.6 to 12.0)	178	11.7 (9.7 to 13.7)	2.6 (2.4 to 2.9)
	≥65	16	6.0 (2.8 to 9.1)	32	6.7 (3.8 to 9.5)	4.6 (3.9 to 5.3)

**Abbreviation:** EAPC = estimated annual percentage change.

* Includes good or better self-rated health, unmet need for mental health services, unemployment, hunger or food security, and housing stability or homelessness.

^†^ Factors were collected in annual data collection cycles that began June 1 of the cycle year and ran through May 30 of the following year.

^§^ All measures self-reported and measured over the previous 12 months except where otherwise noted.

^¶^ Includes 1) sustained viral suppression, defined as all viral load measurements documented undetectable or <200 copies/mL as measured by medical record review; 2) antiretroviral dose adherence, defined as having taken all prescribed antiretroviral doses during the previous 30 days among persons taking antiretroviral therapy; 3) self-reported disability, defined as serious difficulties with hearing, seeing, cognition, mobility, self-care, or independent living; 4) emergency department visit, defined as any visit to an emergency department; and 5) hospitalization, defined as any inpatient hospitalization.

** Includes 1) symptoms of major or other depression, defined as symptoms consistent with a diagnosis of major or other depressive disorder during the previous 2 weeks as measured by the Patient Health Questionnaire-8 among those with any mental health service need and 2) symptoms of generalized anxiety disorder, defined as symptoms consistent with a diagnosis of generalized anxiety disorder during the previous 2 weeks as measured by the Generalized Anxiety Disorder-7 among those with any mental health service need.

^††^ Includes 1) some college education or higher educational attainment, defined as having attended college or having received a college degree and 2) household income at or below poverty threshold, defined as previous calendar year household income at or below poverty threshold according to U.S. Department of Health and Human Services poverty guidelines.

^§§^ Includes 1) unmet need for food assistance or food stamps, defined as needing but not receiving food assistance or food stamps and 2) unmet need for food or meal delivery, defined as needing but not receiving food or meal delivery.

^¶¶^ Includes unmet need for shelter or housing services, defined as needing but not receiving shelter or housing services.

### Unmet Need for Mental Health Services

The 2025 NHAS goal for PWH aged ≥50 years with unmet need for mental health services among those with a need is 9.4%. The observed need in this population was 18.8% (95% CI = 15.4%–22.1%) in 2017 and 21.5% (95% CI = 16.5%–26.5%) in 2022 (Figure 1) (Supplementary Table 1, https://stacks.cdc.gov/view/cdc/160729). The 2025 goal is 56.3% lower than the 2022 estimate. Overall and stratified by age, minimal change in symptoms of major or other depression and symptoms of generalized anxiety disorder among those with a mental health need during 2017–2022 was observed (Table).

### Unemployment

The 2025 NHAS goal for unemployed PWH aged ≥50 years is 5.9%. Unemployment declined from 11.7% (95% CI = 9.7%–13.6%) in 2017 to 8.0% (95% CI = 6.6%–9.5%) in 2022 (Figure 2) (Supplementary Table 1, https://stacks.cdc.gov/view/cdc/160729). The 2025 goal is 26.3% lower than the 2022 estimate. Over time, unemployment was lower among those aged ≥65 years than those aged 50–64 years. Minimal change overall or by age group among factors contributing to unemployment was observed (Table) (Supplementary Table 2, https://stacks.cdc.gov/view/cdc/160728).

### Hunger or Food Insecurity

The 2025 NHAS goal for PWH aged ≥50 years experiencing hunger or food insecurity is 9.0%. Among this population, hunger or food insecurity was 17.9% (95% CI = 15.4%–20.4) in 2017 and 14.1% (95% CI = 12.5%–15.8%) in 2022; those aged ≥65 years experienced the largest reduction in hunger or food insecurity, and this was the only group that met the NHAS 2025 goal by 2022 (Figure 2), (Supplementary Table 1, https://stacks.cdc.gov/view/cdc/160729). The 2025 goal is 36.2% lower than the 2022 estimate for PWH aged ≥50 years. Change in unmet need for food assistance or food stamps was minimal, as was unmet need for food or meal delivery overall and by age group (Table), (Supplementary Table 2, https://stacks.cdc.gov/view/cdc/160728).

### Unstable Housing or Homelessness

The 2025 NHAS goal for PWH aged ≥50 years experiencing unstable housing or homelessness is 7.4%. Unstable housing or homelessness was 14.7% (95% CI = 13.0%–16.4%) in 2018 and 12.5% (95% CI = 10.8%–14.2%) in 2022 (Figure 2) (Supplementary Table 1, https://stacks.cdc.gov/view/cdc/160729). The 2025 goal is 40.8% lower than the 2022 estimate. Over time, except during the 2022 cycle, unstable housing or homelessness was lower among those aged ≥65 years than those aged 50–64 years. Overall and stratified by age, there was little change in unmet need for shelter or housing services during 2017–2022 (Table).

## Discussion

Overall, the five QoL indicators among PWH aged ≥50 years changed little during 2017–2022. QoL estimates among PWH aged ≥65 years were more favorable for unemployment, hunger or food insecurity, and unstable housing or homelessness than among those aged 50–64 years. By 2022, the 2025 goal for decreasing hunger or food insecurity was exceeded among PWH aged ≥65 years. However, for all other indicators and age groups, the magnitude of improvement required to meet 2025 goals suggests these QoL goals will not be met if recent trends continue. The NHAS QoL indicators were adopted in late 2022, leaving <2 years to implement changes to reach 2025 goals (*5*). A federal implementation plan for achieving QoL goals is still being developed (*5*).

Evidence-based interventions exist to improve adherence to antiretroviral therapy, and thus viral suppression^§§^; however, few are tailored to older PWH, who might have specific challenges (e.g., numerous prescribed medications and social isolation).^¶¶^ PWH have poorer physical and mental health than does the overall U.S. population (*7*). Structuring HIV care delivery for older PWH to encompass comprehensive management of chronic diseases and disabilities, including programs that support living with health challenges,*** might improve self-rated health and decrease unmet need for mental health services (*8*). Increasing routine mental health screening and integrating HIV and mental health care could decrease unmet need for these services among PWH (*9*).

Improving QoL and addressing social determinants of health requires a multisectoral approach that moves beyond clinical care. Addressing unemployment can include delivery of skill-building and job-seeking services tailored to older PWH,^†††^ who might face barriers to employment because of age-related disability and discrimination, as well as family caregiving responsibilities. COVID-19–related food and housing challenges resulting from increases in unemployment related to the COVID-19 pandemic, and assistance programs instituted to counteract these challenges, might have affected observed trends.^§§§^ Reductions in unmet need for food assistance might have contributed to meeting the NHAS goal for hunger or food insecurity among PWH aged ≥65 years. Addressing housing insecurity among older PWH might require additional efforts, such as ensuring that federal housing resources are allocated according to need (*10*).

### Limitations

These findings are subject to at least two limitations. First, measurement error might result from recall or social desirability biases, although any biases should not affect assessment of trends if they are constant over time. Second, EAPC is a measure of relative change, so its magnitude is affected by the prevalence of the variable assessed.

### Implications for Public Health Practice

CDC will continue to monitor QoL among PWH to identify areas for intervention. This information can be used to direct multisectoral implementation of programmatic efforts and guide future goals for improving health and well-being among older PWH. CDC-funded HIV prevention and care partners provide linkage to behavioral health and subsistence service providers. The Capacity Building Assistance Program^¶¶¶^ offers technical assistance for addressing social determinants of health, which are closely linked to the NHAS 2025 QoL goals.

## Footnotes

* Good or better self-rated health was defined as reporting one's general health at the time of interview to be good, very good, or excellent as opposed to poor or fair.

† Unmet need for mental health services among those with any need was defined as reporting needing but not receiving services from a mental health professional during the previous 12 months among all persons reporting receiving, or needing but not receiving, services from a mental health professional.

§ Unemployment was defined as reporting being out of work at the time of interview, as opposed to being employed for wages, a homemaker, a student, retired, or unable to work.

¶ Hunger or food insecurity was defined as reporting being hungry and not eating because of lack of money for food during the previous 12 months.

** Unstable housing or homelessness was defined as reporting moving in with others because of financial issues, moving more than two times, being evicted, or living on the street, in a shelter, in a single-room–occupancy hotel, or in a car during the previous 12 months.

†† 45 C.F.R. part 46.102(l)(2), 21 C.F.R. part 56; 42 U.S.C. Sect. 241(d); 5 U.S.C. Sect. 552a; 44 U.S.C. Sect. 3501 et seq.

§§
https://www.cdc.gov/hiv/effective-interventions/treat/index.html

¶¶ https://ryanwhite.hrsa.gov/sites/default/files/ryanwhite/grants/aging-guide-new-elements.pdf (Accessed May 2024).

*** The Chronic Disease Self-Management Program (https://selfmanagementresource.com/programs/small-group/chronic-disease-self-management-small-group/), Positive Self-Management Program (https://selfmanagementresource.com/programs/small-group/hiv-positive-self-management-small-group/), or the Living Well with a Disability program (https://www.cdc.gov/mmwr/volumes/65/su/su6501a10.htm).

†††
https://www.dol.gov/agencies/odep/program-areas/hiv-aids

§§§ For example, although hunger and housing instability might have increased because of pandemic-related job losses and economic impacts, measures like the increase in Supplemental Nutrition Assistance Program benefits, federal eviction moratorium, and authorization for Ryan White HIV/AIDS Program–funded recipients to use Coronavirus Aid, Relief, and Economic Security Act and Emergency Financial Assistance funds to mitigate pandemic-related problems among PWH might have alleviated these increased needs.

¶¶¶
https://www.cdc.gov/hiv/capacity-building-assistance/index.html
